# Supplementary figures and images for: Bridging the Species Gap: Morphological and Molecular Comparison of Feline and Human Intestinal Carcinomas
Source: Cancers (Basel). 2021 Nov 25;13(23):5941. doi: 10.3390/cancers13235941 (PMC8656578; doi:10.3390/cancers13235941)

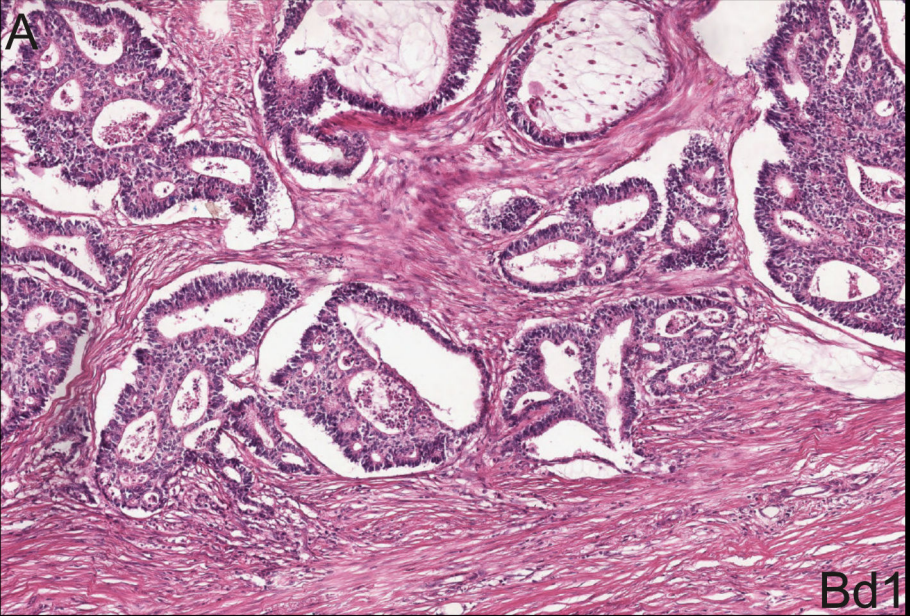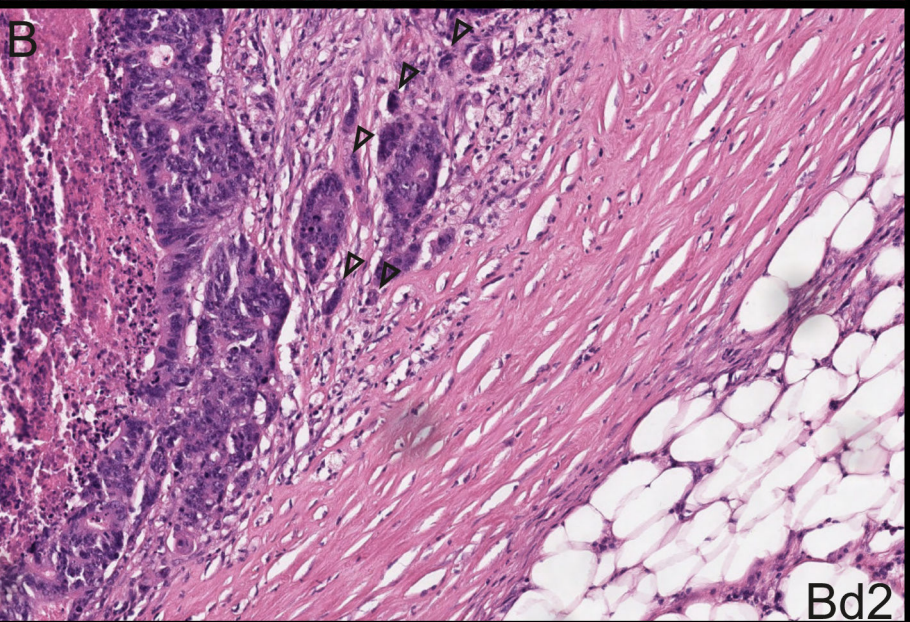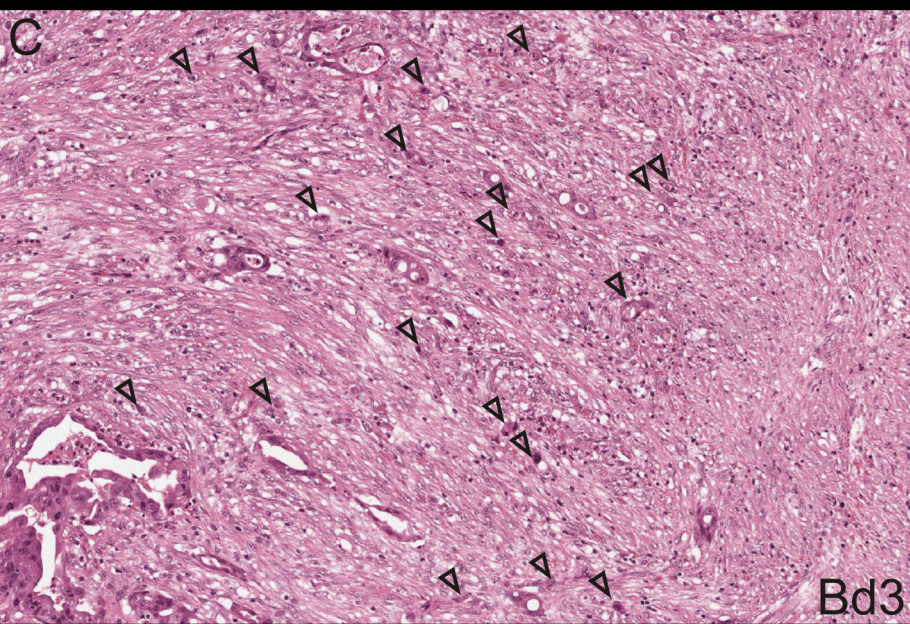

Supplement: Supplementary file 1 [file cancers-13-05941-s001.zip › Supplementary Fig1.pdf]

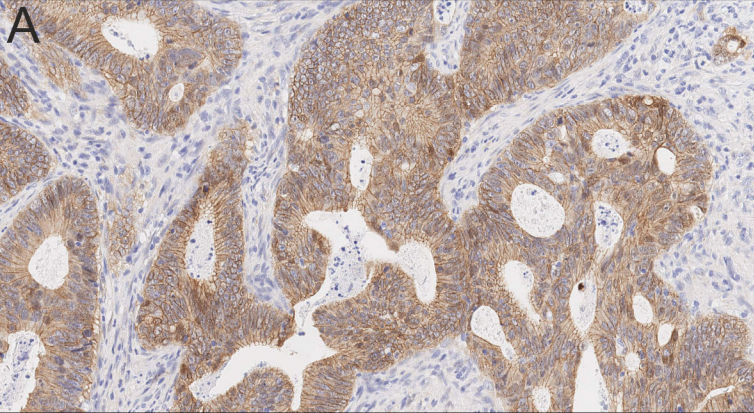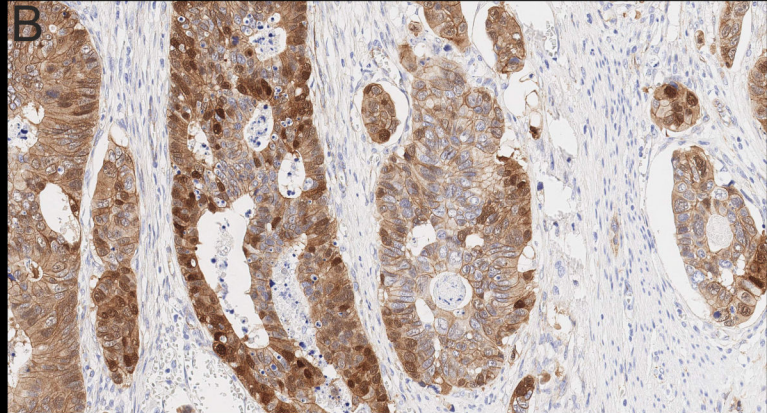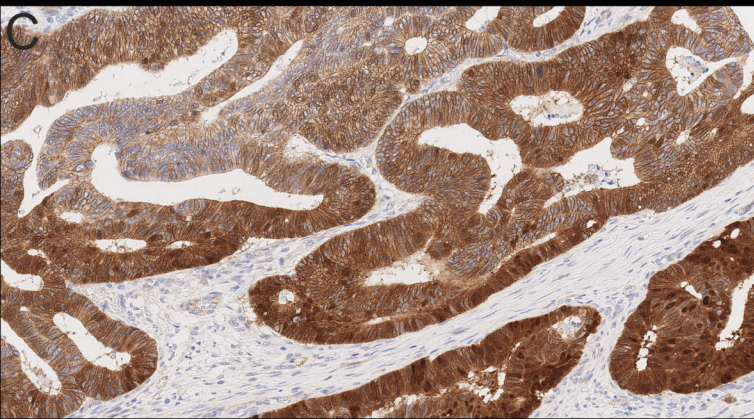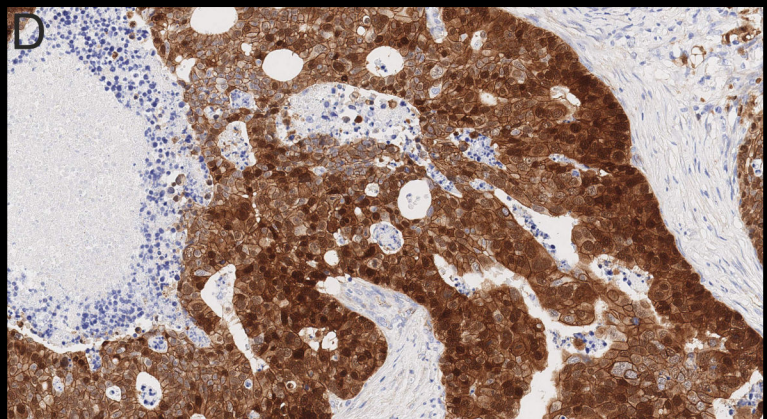

Supplement: Supplementary file 1 [file cancers-13-05941-s001.zip › Supplementary Fig2.pdf]
